# The scenario of delayed graft function in Brazil

**DOI:** 10.1590/2175-8239-JBN-2018-0221

**Published:** 2019-02-25

**Authors:** Tainá Veras de Sandes-Freitas

**Affiliations:** 1 Universidade Federal do Ceará Departamento de Medicina Clínica FortalezaCE Brasil Universidade Federal do Ceará, Departamento de Medicina Clínica, Fortaleza, CE, Brasil.; 2 Hospital Geral de Fortaleza Setor de Transplantes FortalezaCE Brasil Hospital Geral de Fortaleza, Setor de Transplantes, Fortaleza, CE, Brasil.

Brazilian studies report incidences of delayed graft function (DGF) ranging from 54 to 71%^1^^,^^2^, 2- to 3-fold higher than that described in American and European cohorts. The US experienced a recent increase in DGF incidence as a consequence of the Kidney Allocation System (KAS), implemented in December 2014. KAS enabled an increase in the number of kidney transplants (KT) in high-sensitized patients who had been on dialysis for a long time, and resulted in longer cold ischemia time (CIT)^3^. Europe, in turn, has a solid KT program with elderly donors (Eurotransplant Senior Program, ESP). Although donor age is a classic risk factor for DGF, the incidence of this event in KT performed through ESP is about 30%^4^.

In this issue of BJN, Helfer and colleagues^5^ present the results of a retrospective study including 517 deceased donor KT aimed to assess the risk factors for DGF and the impact of its duration on the outcomes. Of note, this study was conducted in a single center with a peculiar feature: a significant number (18%) of KT with organs coming from other Brazilian states, as per the Brazilian laws for organ allocation. This resulted in a high percentage of expanded criteria donors (ECD) and prolonged CIT. The results of this interesting study lead us to reflect on the potential reasons that make transplant centers in Brazil different from European and American centers.

We highlight the incidence of DGF in the cohort: 69.3%. Donor age, final donor creatinine, and CIT were independent risk factors for DGF. In fact, these variables are consistently associated with DGF in previous studies, but some comments are relevant in the Brazilian context.

Mean donor age was 45.7 years in the DGF group. This is similar to or lower than that reported in American and European cohorts. There is no doubt about the impact of age on renal senescence, on the impairment of injury repair mechanisms, and consequently, on the reduction of the ability to deal with the ischemia-reperfusion injury (IRI). However, aging is an inexorable and desirable process worldwide. Thus, age is an unavoidable issue with which we must learn to deal.

Mean final donor creatinine was 1.75 mg/dL in the DGF group. This value is at least 75% higher than that reported by American and European studies, even when considering only elderly donors^4^. Forty-seven percent of the patients were submitted to preimplantation kidney biopsies, but the criteria for accepting organs were not described. It is probable that part of these patients presented renal impairment as a consequence of structural damage resulting from aging and vascular disorders. However, we believe that most donors with renal impairment had acute kidney injury (AKI). In fact, Brazilian studies evaluating deceased donors showed a high percentage of vasoactive drug use, cardiocirculatory arrest episodes, elevated serum sodium and AKI^6^. The evidence supports the hypothesis that inadequate hemodynamic donor maintenance is one of the main reasons for the high DGF incidence in our country.

Mean CIT in the DGF group was 22.5 h. Undoubtedly, CIT is another modifiable variable with significant impact on DGF incidence in Brazil. The main causes for this long time are the large territorial extension, the allocation model, the absence of specific allocation policies for ECD, Complement-dependent citotoxicity (CDC) crossmatch (XM) rather than virtual XM, and CDC-XM using lymphocytes from spleen and lymph nodes obtained only at organ harvesting. Authors did not describe the preservation solutions used, a variable with known impact on DGF incidence, especially in transplants with prolonged CIT.

In line with previous studies, Helfer et al. showed that DGF is associated with acute rejection (AR) episodes (24.5% versus 14.7%) and the combination of DGF and AR is associated with worse renal function and allograft survival^7^. These findings confirm the importance of choosing effective immunosuppressive regimens in patients at high-risk for DGF, including induction therapy with depleting antibodies and surveillance biopsies.

Also aligned with previous data^7^, the study showed that DGF is associated with inferior allograft survival and that the more severe the DGF, the worse the outcomes. Although there is no evidence on the ideal method to assess DGF severity, it is likely that the time until renal function recovery correlates with IRI intensity.

Authors did not observe an impact of DGF on patient survival. These findings contrast with a single-center Brazilian study including 1412 KT, in which prolonged DGF was an independent risk factor for decreased patient survival^7^. The correlation between DGF and mortality seems consistent since these patients receive high-efficacy immunosuppression and have unsatisfactory renal function, known risk factors for cardiovascular events and infections.

This study emphasized the negative impact of DGF on KT outcomes and confirmed DGF risk factors. The data help us target where our efforts should be focused: improving donor maintenance and developing strategies for CIT reduction.
